# Level of job satisfaction and associated factors among rural health extension workers in Hadiya Zone, Southern Ethiopia

**DOI:** 10.1186/s12913-023-09247-4

**Published:** 2023-03-15

**Authors:** Abayneh Tamene, Dansamo Tediso, Achamyelesh Gebretsadik

**Affiliations:** 1 Hadiya Zone Gibe Woreda Health Office, Gibe Woreda, Ethiopia; 2 Department of Public Health, Hawassa College of Health Sciences, Hawassa, Ethiopia; 3 School of Public Health, College of Medicine and Health Science, Hawassa University, Hawassa, Ethiopia

**Keywords:** Job satisfaction, Health extension workers, Hadiya, Ethiopia

## Abstract

**Background:**

The improvement of various health outcomes, including the reduction of maternal, neonatal, infant, and child mortality as well as the increased use of maternity and newborn health services, was significantly assisted by rural health extension workers. Implementing health extension programs and offering the community high-quality healthcare services requires job satisfaction. In the study area, there has been little research on job satisfaction and related variables. Therefore, the purpose of this study was to evaluate the degree of job satisfaction among rural health extension workers in the Hadiya zone, Southern Ethiopia, as well as associated factors.

**Methods:**

A facility-based cross-sectional study was conducted among 262 rural health extension workers from May 30, 2021, to July 02, 2021. A simple random sampling method was used to select six rural districts. Data were gathered utilizing structured interviewer-administered questions and skilled data collectors. EpiData V4.4.1 was used to store the data, and SPSS Version 25 was used for analysis. To determine the relationship between the variables, bivariate and multivariate logistic regressions were used. The association was reported using the adjusted odds ratio (AOR) with a 95% confidence interval (95%CI), and the significance level was set at a p-value of 0.05.

**Results:**

This study showed that 52.7% of rural health extension workers were satisfied with their jobs. Support from Keble leaders [AOR = 5.3; 95% CI (2.6, 11.1)], on-job training [AOR = 5.7, 95% CI (2.2, 14.9)], supportive supervision [AOR = 4.3; 95% CI (1.7, 10.8)] and reward or recognition [AOR = 7.4, 95% CI (3.0, 18.1)] were factors that associated with job satisfaction of health extension workers.

**Conclusion:**

According to this study, more than half of rural health extension workers were happy with their jobs. Health extension workers’ job satisfaction was affected by support from keble leaders, supportive supervision, on-the-job training, and recognition and rewards. In order to increase the happiness of the health extension workers, supporting supervision must be strengthened and the best performers must be recognized.

**Supplementary Information:**

The online version contains supplementary material available at 10.1186/s12913-023-09247-4.

## Background

According to the World Health Organization report, by 2035, South East Asia and Africa will suffer 12.9 million healthcare professional losses. This survey also highlighted the fact that due to insufficient incentives and inadequate compensation, almost 40% of health professionals globally will leave their jobs in the upcoming decades [1].

Ethiopia is one of several nations impacted by the lack of health workforce density, particularly the shortage of doctors, nurses, and midwives, which is well below the minimum threshold density of 2.3 health workers per 1000 people for countries to achieve critical services [2].

Additionally, most health professionals tend to be dissatisfied with the majority of their jobs for a variety of reasons [3]. In Ethiopia, Health Extension Workers (HEWs) represent the majority of the healthcare workforce and have significantly increased the availability of healthcare workers, especially in rural areas [4].

As part of the second five-year Health Sector Development Plan, Ethiopia established the Health Extension Program (HEP) in 2004 [5]. To reduce the high turnover of health professionals, one of the main strategies in the design of HEP is the use of female HEWs who are recruited from respective areas and deployed [6]. One functioning health post allows two HEWs to serve 5,000 people inside a kebele. After completing technical and vocational training, the HEWs are salaried healthcare professionals who are assigned at Health Post (HP) level to deliver curative, promotive, and preventive services.

Since the HEP’s national rollout, they have significantly improved a number of health outcomes, including a decline in maternal, neonatal, infant, and child mortality and an increase in the use of maternal and newborn health services [7].

To implement HEPs and deliver high-quality healthcare to the community, the job satisfaction of the health extension worker is crucial [6, 8]. Their poor performance and increased likelihood of leaving their jobs are both caused by their job dissatisfaction [9]. This was evident at the time due to the high turnover rate among HEWs and the frequent closing or merging of health posts [9]. For instance, a study conducted in the Illubabora zone revealed that overall HEW turnover intention was 52%, indicating that there may not be enough HEWs available for broad activity, which makes it challenging to deliver all necessary services and address the needs of the entire community [10].

According to the studies that have already been done, factors that affect job satisfaction include low salaries, poor supervision, low levels of personal recognition, an inadequate career structure, an unattractive salary scale, and a lack of basic infrastructures like electricity and water supply in the workplace [8, 11]. Low levels of motivation and job satisfaction among health workers, particularly in the areas of access to further training and promotion and poor physical working conditions, contributed to performance-related issues such as ineffective patient care, nonresponse to patient needs, and absenteeism [12, 13]. However, other equally important factors like transfer opportunity, distance from their home, and workload of HEWs have been given little attention, and thus were not well addressed by other studies.

The question of the degree of job satisfaction and its determinants among health professionals has been the subject of numerous types of research throughout the world and in Ethiopia. However, the majority of them concentrate on the satisfaction of professionals, such as nurses, midwives, or doctors working in hospitals and health facilities [14–16]. Due to this, little is known about rural HEWs’ job satisfaction despite their difficult working conditions and significant contributions to the rural community [9, 17]. To the best of our knowledge, there were few published studies related to job satisfaction among rural HEWs, except in the East Shoa zone Oromia Region, Gambella Region, and Sidama regional state [8, 11–18]. So far, no study has been conducted in Southern Nations Nationalities and Peoples Region (SNNPR) particularly, in the study area.

The finding of this study may provide the data for Zonal Health Department, Regional Health Bureau, and policymakers to design intervention strategies that can satisfy and motivate HEWs. Satisfied and motivated HEWs are important for better performance, improvement of health coverage, better delivery of services, and undertaking the necessary actions to strengthen the health extension program. Therefore, this study aimed to assess the job satisfaction of HEWs and associated factors in the Hadiya Zone, Southern Ethiopia.

## Methods

### Study area

This study was conducted in the Hadiya zone, which is found in Southern Nations Nationalities and Peoples Region (SNNPR). It is located 230 km from Addis Ababa capital city of Ethiopia, and 178 km from Hawassa. The zone is divided into 12 rural and seven urban districts. According to the 2020 Hadiya zone health department report, the total population of rural districts is 1,737,796. Among these 884,538 (50.9%) are females, 404,906 (23.3%) are reproductive age group (15–49 years) and 271,096 (15.6%) are under five ages. There is one government comprehensive specialized teaching and referral hospital, three primary hospitals, 61 health centers, and 310 health posts. The total health workforce of the zone without supportive staff was 3123, of which 633 (20%) of the zone health workforce are rural health extension workers [19].

### Study design, period, and population

A facility-based cross-sectional study was conducted from May 30, 2021, to July 02, 2021. The source population was all rural health extension workers working in the Hadiya zone. The study population was all eligible rural health extension workers who have served for at least six months in the selected districts. Those HEWs who were seriously ill during the data collection period were excluded from this study.

### Sample size determination and sampling technique

The sample size was determined by using a single population proportion formula in consideration of the following assumption: − 95% confidence level, 4% margin of error, and the estimated proportion of rural health extension workers satisfied with their job in the East Shoa zone, Oromia, Ethiopia was 18% [11], and 10% non-response rate. The determined sample size in the final was 251. Due to the close proximity of the calculated sample size and the overall number of health extension workers in the study area, all rural health extension workers (262) employed in the health posts located in the chosen districts were included in the study.

### Data collection procedures

A standardized questionnaire that was administered by an interviewer was used to gather the data. Based on the Minnesota satisfaction survey [20], we changed the outcome variable to fit the study area’s context. Data were gathered by two female midwives and four male nurses with diplomas who are fluent in Hadiyisa and Amharic and have previous expertise in data collecting. The data gathering was additionally supervised by two first-degree public health professionals. The information was gathered during each catchment’s weekly command post-meeting, which is led by HEWs.

### Data quality control

To ensure uniformity, the questionnaire was written in English, translated into Amharic, and then returned to English. Before the actual data collection period outside the chosen areas, the pre-test was conducted using 5% of the sample size. The principal investigator reviewed the completed questionnaires after the data collectors and supervisors had received a two-day training session on the study’s objectives, how to handle questionnaires, how to collect data, and ethical considerations. Daily and weekly checks were made by the principal investigator for missing values and data completeness by the supervisor.

### Study variables

Independent variables were: socio-demographic (age, marital status, education level, recruited address, residence, service in a year), infrastructure and work environment-related factors, salary and other benefit-related factors, management-related factors, and workload-related factors.

The dependent variable, job satisfaction, was measured with the help of 16 items by taking into account various job satisfaction scale items’ dimensions. Each item has a Likert scale of 1 (very dissatisfied) to 5 (extremely satisfied). The items were created by studying various academic works, and the total job satisfaction score was calculated by averaging all the scale’s items’ scores [15, 21, 22]. Each person’s level of job satisfaction was measured individually using the overall satisfaction level, with the mean of all subscales serving as the cutoff value. As a result, health extension workers were classified as “satisfied” or “dissatisfied” depending on whether their score was above or below the mean [11, 21, 23].

### Operational definitions

On-job training: Any form of formal skill-developing short-term training given by the district health office, Zonal health department, Regional Health Bureau, Federal Ministry of Health, or in collaboration with any stakeholder.

Working time per week: Related to the amount of working time in that working hour of HEW per week are more than 39 h was considered a high, and if it is less than or equal to 39 h per week, it was considered as low.

Transfer: Changing workplace (kebele ) of HEWs, usually done annually by public service and woreda health offices based on the request of HEWs and some predefined rules.

Recognition: the act of appreciation and acknowledgment of HEWs for their performance by the catchment health center, district health office, Zonal Health Department, or other concerned bodies, and it may be verbal praise or giving a gift or reward.

Support: any actions that motivate, strengthen, and encourage health extension workers to promote their performance and to ensure the delivery of quality health services, which is given from the catchment health center, district health office, zonal health department, regional health bureau, or other non-governmental organization (NGO).

### Data processing and analysis

Data were cleaned, coded, and entered into Epi Data version 4.4.1 and analyzed using SPSS version 25. Descriptive analysis was used to summarize the data. Bivariable logistic regression analysis was done to identify the statistical association between each independent and dependent variable. The maximum variance inflation factor (VIF) for all independent variables was 5.9. Variables with a P-value < 0.25 in the bivariable logistic regression analysis were considered for multivariable logistic regression analysis. Adjusted odds ratio with 95% CI was calculated to determine the presence and strength of association among predictors and outcome variables. Finally, we consider the p-values less than 0.05 as statistically significant.

## Results

### Socio-demographic characteristics of health extension workers

The study included 262 rural HEWs in total, with a response rate of 100%. The average age of HEWs is 29.2 years (SD: 3.3), with a range of 20 to 38 years. The age range of 26 to 30 years was represented by more than half (53.1%) of the respondents. The majority of the research participants (84.7%) were married. Regarding educational attainment, 76% of respondents had a college degree or higher (level IV), and roughly 45.8% had been served for six to ten years. Most respondents (84.9%) were chosen from their working kebeles, while nearly three-fourths (74.4%) of the respondents lived outside of their working kebeles [Table 1].

**Table 1:** Socio-demographic characteristics of HEWS in Hadiya Zone, Southern Ethiopia, 2021. (N = 262)

Variables	Category	Frequency(n)	Percent (%)
Age	20–25	31	11.8
	26–30	139	53.1
	≥ 31	92	35.1
Educational level	Certificate/level III	63	24
	College diploma and above/ level IV	199	76
Marital status	Single	37	14.1
	Married	222	84.7
	Others (Divorced & Widowed)	3	1.1
Working time	≤ 5	26	9.9
	6–10	120	45.8
	> 10	116	44.3
Residence	In the working kebeles	67	25.6
	Outside working kebeles	195	74.4
Recruited address	From working kebeles	225	85.9
	Outside the working kebeles	37	14.1

### Infrastructures and work environment

In terms of infrastructure and factors affecting the workplace, respectively, 92%, 99.2%, and 66% of HEWs work in areas without access to water, power, or transportation. 76% of the population in the working kebele lacks housing services. 97.3% of HEWs said they received recognition from the community, and 69.5% said their kebele leaders gave them support [Table 2].

**Table 2:** Infrastructures and work environment characteristics of HEWs in Hadiya Zone, Southern Ethiopia, 2021. (N = 262)

Variables	Category	Frequency(n)	Percent (%)
Water supply	Yes	21	8
	No	241	92
Availability of electricity	Yes	2	0.8
	No	260	99.2
Transport access	Yes	89	34
	No	173	66
Housing service	Yes	63	24
	No	199	76
Availability of medical equipment	Yes	243	92.7
	No	19	7.3
Support from kebele leaders	Yes	182	69.5
	No	80	30.5
Community recognition	Yes	255	97.3
	No	7	2.7

### Salary and other benefit characteristics

Only 14.1% of HEWs have a monthly wage of less than or equal to 5000 Ethiopian Birrs (94.8 US$), whereas more than half (65.6%) have more than 7000 Ethiopian Birrs (132.8 US$). The majority of HEWs (80.2%) got on-the-job training to increase the effectiveness and efficiency of their work. The majority of HEWs (80.5%) received educational opportunities, and most (66.4%) of them were promoted equitably and at the appropriate time [Table 3].

**Table 3:** Salary and other benefit related characteristics of HEWs in Hadiya Zone, Southern Ethiopia, 2021. (N = 262)

Variables	Category	Frequency(n)	Percent (%)
Salary	≤ 5000 ETB (≤ 94.8 US$)	37	14.1
	5001-7000ETB/94.85–132.8 US$	53	20.2
	≥ 7001ETB/132.85 US$	172	65.5
On job training	Yes	210	80.2
	No	52	19.8
Educational opportunity/upgrading/	Yes	211	80.5
	No	51	19.5
Promotion	Yes	174	66.4
	No	88	33.6

### Management characteristics

When it comes to supportive supervision, the majority of HEWs (80.5%) said that it was carried out within 6 months from their superiors. The majority of HEWs (90.5%) did not have the opportunity for workplace transfer, and nearly three-fourths (73.7%) of HEWs did not receive any recognition for their efforts [Table 4].

**Table 4:** Management related characteristics of HEWs in Hadiya Zone, Southern Ethiopia, 2021. (N = 262)

Variables	Category	Frequency(n)	Percent (%)
Supportive supervision within	Yes	211	80.5
last 6 months	No	51	19.5
Any reward or recognition	Yes	69	26.3
	No	193	73.7
Chance of work place transfer	Yes	25	9.5
	No	237	90.5

### Working hour

Most (84.7%) HEWs claimed that their kebeles had a population of above 5000, and about (69.1%) reported that they work more than thirty-nine hours each week. More than half (63%) of kebeles were located more than ten kilometers from the woreda town, and almost 48.9% of HEWs traveled six to ten kilometers to get to the health post from their home. The number of HEWs was one in 16.4% of the kebeles [Table 5].

**Table 5:** Working hour of HEWs in Hadiya Zone, Southern Ethiopia, 2021. (N = 262)

Variables	Category	Frequency(n)	Percent (%)
Working hour per week	≤ 39	81	30.9
	> 39	181	69.1
Total population of kebeles	≤ 5000	40	15.3
	> 5000	222	84.7
Distance of woreda town from kebeles	≤ 5Km	8	3.1
	6–10 Km	89	34
	> 10 Km	165	63
Distance of kebele from home	≤ 5Km	91	34.7
	6–10 Km	128	48.9
	> 10 Km	43	16.4
Number of HEWs in the kebeles	1	43	16.4
	2	205	78.2
	≥ 3	14	5.4

### Level of job satisfaction

Rural HEWs in the research area reported an average job satisfaction level of 52.7% (95% CI: 47.3, 58). In terms of satisfaction, the majority of HEWs (84.7%) expressed satisfaction with their work as HEWs, and 69.5% expressed happiness with assisting the community. On the other side, 36.2% of the respondents expressed dissatisfaction with the accessibility of medical supplies and equipment, while 70.2% of HEWs expressed dissatisfaction with the acknowledgment or compensation for their work from superiors. Only 26.3% of HEWs said they were happy with the supportive supervision from their supervisors, and only 21.3% said they were happy with the help from their kebele leaders [Table 6].

**Table 6:** Sixteen subscales of job satisfaction of HEWs in Hadiya Zone, Southern Ethiopia, 2021. (N = 262)

Satisfaction	Respondents’ response to satisfaction questions				
	Very satisfied	Satisfied	Neutral	Dissatisfied	Very dissatisfied
Your current salary	0(0%)	43(16.4%)	136(51.9%)	79(30.2%)	4(1.5%)
Career development	2(0.8%)	56(21.4%)	112(42.7%)	89(34)	3(1.1%)
Job/short term training	13(5%)	114(43.5%)	77(29.4%)	45(17.2%)	13(5%)
Educational opportunity/upgrading	4(1.4%)	96(36.6%)	92(35.1%)	59(22.5%)	11(4.2%)
Your working place(kebele)	2(0.8%)	64(24.4%)	117(44.7%)	76(29%)	3(1.1%)
Availability of medical equipment’s/supplies	3(1.1%)	31(11.8%)	133(50.8%)	92(35.1%)	3(1.1%)
Helping the community	69(26.3%)	153(58.4%)	32(12.2%)	8(3.1%)	0(0%)
Relationship with colleague HEWs	28(10.7)	143(54.6%)	76(29%)	14(5.3%)	1(0.4%)
Support from your kebele leaders for your work	3(1.1%)	53(20.2)	80(30.5%)	88(33.6%)	38(14.5%)
Relationship with Health center staff	6(2.3%)	99(37.8%)	133(50.8%)	23(8.8%)	1(0.4%)
Relationship with Woreda health office staff	4(1.5%)	87(33.2%)	143(54.6%)	26(9.9%)	2(0.8%)
Supportive supervision from your supervisors	4(1.5%)	65(24.8%)	74(28.2%)	82(31.3%)	37(14.1%)
Recognition/reward for your work from your superiors	1(0.4%)	45(17.2%)	32(12.2%)	108(41.2%)	76(29%)
Technical support from the health center	4(1.5%)	90(34.4%)	75(28.6)	72(27.5%)	21(8%)
Time you have for your family	1(0.4%)	21(8%)	90(34.4%)	143(54.6%)	7(2.7%)
Working as HEW	44(16.8%)	138(52.7%)	72(27.5%)	8(3.1%)	0(0%)

### Factors associated with job satisfaction

In multivariable logistic regression analysis, supported by kebele leaders, on-job training, supportive supervision within the last 6 months, and reward or recognition showed a significant association with job satisfaction.

Accordingly, HEWs who received support for their work from their kebele leaders were 5.3 times more likely to be satisfied than those who didn’t [AOR = 5.3; 95% CI (2.6, 11.1)]. Similar to this, HEWs were 4.3 times more likely to be satisfied with their jobs than those who were not monitored by their superiors within the previous six months [AOR = 4.3; 95% CI (1.7,10.8)]. In addition, HEWs who had the opportunity for on-the-job training during the previous year were 5.7 times more likely to be satisfied with their jobs than those who did not [AOR = 5.7,95% CI (2.2,14.9)]. Additionally, when compared to HEWs who did not get any awards or recognition for their achievement, those who did were 7.4 times more likely to be satisfied with their jobs [AOR = 7.4, 95% CI (3.0,18.1)] [Table: 7].

**Table 7:** Factors associated with job satisfaction of rural HEWs in Hadiya Zone, Southern Ethiopia, 2021. (N = 262)

Variables	Job Satisfaction		Bivariate analysis Crude OR [95% CI]	Multivariate analysis Adjusted OR [95% CI]
	Satisfied	Dissatisfied		
Educational level				
Certificate/level III	25 (39.7%)	38 (60.3%)	1	1
College diploma and above/ level IV	113 (56.8%)	86 (43.2%)	2 (1.1,3.6) *	1 (0.3,3.2)
Residence				
In the kebele	44 (65.7%)	23 (34.3%)	2.1 (1.2,3.7) *	0.2 (0.04,1.4)
Outside the kebele	94 (48.2%)	101 (51.8%)	1	1
Service in year				
≤ 5 Years	11 (42.3%)	15 (57.7%)	1	1
6–10 Years	59 (49.2%)	61 (50.8%)	1.3 (0.6,3.1)	1.2 (0.4,3.1)
> 10 Years	68 (58.6%)	48 (41.4%)	1.9 (0.82,4.57)	2.4 (0.9,6.6)
Water supply				
Yes	16 (76.2%)	5 (23.8%)	3.1 (1.1,8.8) *	0.8 (0.2,2.8)
No	122 (50.6%)	119 (49.4%)	1	1
Transport service				
Yes	55 (61.8%)	34 (38.2%)	1.8 (1.04,2.95) *	0.9 (0.5,1.9)
No	83 (48%)	90 (52%)	1	1
Housing service in the kebele				
Yes	44 (69.8%)	19 (30.2%)	2.6 (1.41,4.74) *	3.4 (0.5,22.8)
No	94 (47.2%)	105 (52.8%)	1	1
Support from kebele leaders				
Yes	122 (67%)	60 (33%)	8.1 (4.3,15.2) **	5.3 (2.6,11.1) **
No	16 (20%)	64 (80%)	1	1
Monthly salary				
≤ 5000ETB(≤ 94.8 US$)	15 (40.5%)	22 (59.5%)	1	1
5001-7000ETB/94.85–132.8 US$	22 (41.8%)	31 (58.5%)	1.0 (0.4,2.4)	0.7 (0.16,3.0)
≥ 7001ETB/132.85 US$	101 (58.7%)	71 (41.3%)	2.08 (1.01,4.3) *	0.72 (0.2,3.5)
On job training				
Yes	125 (59.5%)	85 (40.5%)	4.4 (2.22,8.8) **	5.7 (2.2,14.9) **
No	13 (25%)	39 (75%)	1	1
Supportive supervision				
Yes	130 (61.6%)	81 (38.4%)	8.6 (3.9,19.3) **	4.3 (1.7,10.8) *
No	8 (15.7%)	43 (84.3%)	1	1
Reward or recognition				
Yes	59 (85.5%)	10 (14.5%)	8.5 (4.1,17.7) **	7.4 (3.0,18.1) **
No	79 (40.9%)	114 (59.1%)	1	1
Distance of kebele from home				
≤ 5 Km	64 (70.3%)	27 (29.7%)	4.9 (2.3,10.7) **	2.3 (0.7,7.9)
6–10 Km	60 (46.9%)	68 (53.1%)	1.8 (0.88,3.78)	1.27 (0.5,3.3)
> 10 Km	14 (32.6%)	29 (67.4%)	1	1

*-P value less than 0.05, **-P value less than 0.001

## Discussion

According to this study, 52.7% of rural HEWs reported being generally satisfied with their jobs. This result was equivalent to those of studies conducted in Addis Ababa, Ethiopia (52, 9%) [16], northwest Ethiopia (54%) [24], South Africa (52.1%) [25], and India (50%) [26]. However, our results fell short of the satisfaction rates reported in Tanzania (82.6%) and Malawi (71%), respectively [25]. This discrepancy might result from variations in the socioeconomic characteristics, work environment, and organizational structure of a healthcare professional. Our results, however, were greater than those of research conducted in the Harari region (44.2%) [23], West Ethiopia (41.46%) [21], Amhara Region (31.7%) [22], Sidama (36.6%) [27], Eastern Ethiopia (38.5%) [28], Sri Lanka (23.7%) [29], Pakistan (41%) [30], and Turkey (45.5%) [31]. This fluctuation may be caused by variations in the sociodemographic characteristics of the study population, the study environment, and the study period. The different instruments used to measure the satisfaction level could be another cause for the above discrepancy.

HEWs who received support from kebele leaders for their work were more satisfied with their jobs than their counterparts in terms of factors related to job satisfaction. This may be since the HEWs work other sector’s in addition to the health sector. As a result, the kebele leaders’ support lessens the heavy workload they must bear, which may contribute to greater job satisfaction. An investigation carried out in the Illubabora Zone, South West Ethiopia [10], the North Wollo Zone, Northeast Ethiopia [9], Switzerland [32], and Tanzania [33] all provided evidence in support of this conclusion.

According to this study, HEWs who had the opportunity to receive on-the-job training during the previous year reported higher levels of job satisfaction than their counterparts. This result is consistent with findings from the Oromia Region [34], the North Wollo zone, northeast Ethiopia [9], Kenya [35], Nigeria [36], and Pakistan [30] as well as the west Shoa zone. This could be explained by the fact that training boosts HEWs’ self-confidence and self-esteem and enhances the quality of care, both of which would significantly increase their motivation and satisfaction [34]. Additionally, various studies have shown that the health sector’s human resources require ongoing training and refresher courses.

The HEWs who had supportive supervision from their superiors within the last six months reported higher job satisfaction than their counterparts. This result is similar with research done in rural Papua New Guinea among rural health workers, in Gondar Referral Hospital among midwives, and in Addis Ababa city among midwives, where supportive supervision was found to positively improve job satisfaction of health professionals [16, 22, 37]. Additionally, in the Gambella Region, inadequate supervision was cited as a factor in rural HEWs’ dissatisfaction and demotivation [8]. This may be due to supportive supervision, which makes HEWs enjoy their workplace and perform better, which in turn motivates and boosts job satisfaction.

Furthermore, HEWs who received incentives or recognition for their work were more satisfied in their positions than their competitors. This result was consistent with research done in the Eastern Cape [38], Kenya [39], and East Shoa Zone [8, 15] in Ethiopia, as well as North Wollo [9], Gambella, and Iran [40]. This might be explained by saying that praise and prizes may encourage HEWs to work harder, develop new abilities, develop a good attitude toward achieving organizational goals, and lastly, this might lead to job satisfaction.

### Limitation the study

The purpose of our study was to evaluate the degree of job satisfaction and contributing factors among rural HEWs in the zone. Despite this, this study had certain limitations that should be taken into account when interpreting the findings. There were no qualitative methods used in this study to evaluate rural HEWs’ job satisfaction. Additionally, because our study only included rural HEWs, generalizing the findings to all zone employees may not be viable. Furthermore, because the study was cross-sectional, it was challenging to pinpoint the causal-effect connections between job satisfaction and related factors.

## Conclusion

In comparison to the combined prevalence of job satisfaction among Ethiopian healthcare professionals, this survey found that more than half of rural HEWs had good job satisfaction. On-the-job training, supportive supervision, kebele leader assistance, and rewards or recognition were found to be strongly linked determinants with job satisfaction. As a result, the regional Health Bureau and its partners ought to provide for HEWs to get crucial short-term training opportunities. The district health offices and the Hadiya zone health department should focus more on inspiring HEWs by offering various awards and recognition. Together with the district health offices and the staff of the catchment health centers, the Hadiya zone health department should increase the adequate and encouraging supervision of HEWs.

## Electronic supplementary material

Below is the link to the electronic supplementary material.

Supplementary Material 1

## Data Availability

The datasets used and/or analyzed during the current study available from the corresponding author on reasonable request.

## Abbreviations

ANC: Antenatal care
HEP: Health Extension Programme
HES: Health Extension Service
HEWs: Health Extension Workers
HP: Health Post
HSDP: Health Sector Development Plan
MCH: Maternal and Child Health
NGOs: Non-Governmental Organizations
PHC: Primary Health Care
PNC: Post Natal Care
SDG: Sustainable Development Goal
SNNPR: Southern Nations Nationalities and Peoples Region
SPSS: Statistical Package for Social Science
UHEP: Urban Health Extension Programme
VIF: Variance Inflation Factor
