# Supplementary material for: Level of job satisfaction and associated factors among rural health extension workers in Hadiya Zone, Southern Ethiopia
Source: BMC Health Serv Res. 2023 Mar 15;23:258. doi: 10.1186/s12913-023-09247-4 (PMC10018938; doi:10.1186/s12913-023-09247-4)
Supplement: Supplementary file 1 — Supplementary Material 1 [file 12913_2023_9247_MOESM1_ESM.docx]

## Annex : English Version Questionnaire

**Hawassa University College of Medicine and Health Sciences School of Public Health**.

**Title: assess job satisfaction and associated factors among rural health extension workers in Hadiya Zone, Southern Ethiopia.**

Questionnaire code number

Name of the data collector Date of interview Signature Name of the supervisor Date of checking Signature

**Part One: Socio demographic factors**

| S.No | Questions | Response options / Codes | Skip |
| --- | --- | --- | --- |
| 101 | What is your age now? | years |  |
| 102 | What is your educational level? | 1. Level III 2. Level IV |  |
| 103 | What is your current marital status? | 1. Single 2. Married 3. Divorced 4. Widowed |  |
| 104 | Place of your residence/home/? | 1. In the kebele that I ‘m working 2. Outside the working kebele |  |
| 105 | Recruited address | 1. From working kebele 2. Outside the working kebele |  |
| 106 | Working time |  |  |

**Part two**: **Infrastructures and work environment related factors**

| 201 | Water supply available in the HP? | 1. Yes 2. No |  |
| --- | --- | --- | --- |
| 202 | Availability of electricity? | 1. Yes 2. No |  |
| 203 | Accessibility of transport service? | 1. Yes 2. No |  |
| 204 | Availability of housing service in working kebele? | 1. Yes 2. No | If 2,  skip to Q 206 |
| 205 | If ‘Yes’ for Q 204, house ownership | 1. Provided by kebele 2. Rented 3. Own |  |
| 206 | Availability of medical equipment’s/supplies? | 1. Yes 2. No |  |
| 207 | Do you get any support from your kebele leaders for your work? | 1. Yes 2. No |  |
| 208 | Does the community recognize your work? | 1. Yes 2. No |  |

**Part three: Salary and other benefit factors**

| 301 | Your current monthly salary | ETB |  |
| --- | --- | --- | --- |
| 302 | Have you got chance of on job training within the last 1 year? | 1. Yes 2. No |  |
| 303 | Have you ever got further education opportunity/upgrading? | 1. Yes 2. No |  |
| 304 | Do you get your career growth fairly and at right time? | 1. Yes 2. No |  |

**Part four: Management related factors**

| 401 | Have you ever received any supportive supervision with in last 6 months? | 1. Yes 2. No | | | | If 2,  skip to Q404 |
| --- | --- | --- | --- | --- | --- | --- |
| 402 | If “Yes” for Q 401, from whom? | 1. Health center 2. Woreda Health office 3. Zonal Health Department 4. Regional Health Bureau 5. NGO 6. Other specify_ _ | | | |  |
| 403 | Frequency of supervision | Health center | 1 | 2 | ≥3 |  |
|  |  | Woreda Health office | 1 | 2 | ≥3 |  |
|  |  | Zonal health Department | 1 | 2 | ≥3 |  |
|  |  | Regional Health Bureau | 1 | 2 | ≥3 |  |
|  |  | Other NGO | 1 | 2 | ≥3 |  |
| 404 | Have you ever received any reward or recognition for your work? | 1. Yes 2. No | | | | If 2,  skip to Q406 |
| 405 | If your answer is “Yes” for question 405, from whom did you got the reward or recognition? | 1. Catchment health center 2. Woreda health office 3. Zonal health department 4. Other   specify | | | |  |
| 406 | Have you ever got chance of work place transfer after you are assigned your first place of work? | 1. Yes 2. No | | | |  |

**Part five: Working hour related factors**

| 501 | Working hour/week | Hours |  |
| --- | --- | --- | --- |
| 502 | What is the total population in your kebele |  |  |
| 503 | What is the distance of your kebele from the woreda town? | KM |  |
| 504 | What is the distance of your kebele from your home? | KM |  |
| 505 | What is (are) the number of HEWs working in your kebele? |  |  |

**Part six**: **Job satisfaction questionnaire**

Now I want to ask how you feel about your current job. Please tell me your satisfaction level for each statement using a 5-point scale.

1. If the question gives you more than you expected, circle 5 under ‘’very satisfied’’
2. If the question gives you what you expected, circle 4 under ‘’satisfied’’
3. If you cannot make up your mind whether or not the question gives you what you expected,

circle 3 under ‘’Neutral’’

1. If the question gives you less than you expected, circle 2 under ‘’dissatisfied’’
2. If the question gives you much less than you expected, circle 1 under ‘’very dissatisfied’’

| S.No | Please circle the appropriate answer in the box | Very dissatisfied | Dissatisfied ed | Unable to decide  (Neutral) | Satisfied | Very satisfied |
| --- | --- | --- | --- | --- | --- | --- |
| 601 | Satisfaction on your current salary | 1 | 2 | 3 | 4 | 5 |
| 602 | Satisfaction on career development | 1 | 2 | 3 | 4 | 5 |
| 603 | Satisfaction in on job/ short term  training | 1 | 2 | 3 | 4 | 5 |
| 604 | Satisfaction with educational  opportunity /upgrading | 1 | 2 | 3 | 4 | 5 |
| 605 | Satisfaction on your working place  (kebele) | 1 | 2 | 3 | 4 | 5 |
| 606 | Availability of medical  equipment’s/supplies | 1 | 2 | 3 | 4 | 5 |
| 607 | Satisfaction on helping the  community | 1 | 2 | 3 | 4 | 5 |
| 608 | Satisfaction on relationship with  colleague HEWs | 1 | 2 | 3 | 4 | 5 |
| 609 | Satisfaction on support from your  kebele leaders for your work | 1 | 2 | 3 | 4 | 5 |

| 610 | Satisfaction on relationship with  Health center staff | 1 | 2 | 3 | 4 | 5 |
| --- | --- | --- | --- | --- | --- | --- |
| 611 | Satisfaction on relationship with  Woreda health office staff | 1 | 2 | 3 | 4 | 5 |
| 612 | Satisfaction on supportive  supervision from your supervisors | 1 | 2 | 3 | 4 | 5 |
| 613 | Satisfaction on recognition/reward  for your work from your superiors | 1 | 2 | 3 | 4 | 5 |
| 614 | Satisfaction on technical support  from the health center | 1 | 2 | 3 | 4 | 5 |
| 615 | Satisfaction on time you have for  your family | 1 | 2 | 3 | 4 | 5 |
| 616 | Satisfaction on working as HEW | 1 | 2 | 3 | 4 | 5 |

Thank you for your cooperativeness!!!

Cheeked by supervisor/investigator/ Signature
